# The active free-living bathypelagic microbiome is largely dominated by rare surface taxa

**DOI:** 10.1093/ismeco/ycae015

**Published:** 2024-01-23

**Authors:** Marta Sebastián, Caterina R Giner, Vanessa Balagué, Markel Gómez-Letona, Ramon Massana, Ramiro Logares, Carlos M Duarte, Josep M Gasol

**Affiliations:** Department of Marine Biology and Oceanography, Institut de Ciències del Mar, CSIC. Pg Marítim de la Barceloneta 37-49, Barcelona, Catalunya E08003, Spain; Department of Marine Biology and Oceanography, Institut de Ciències del Mar, CSIC. Pg Marítim de la Barceloneta 37-49, Barcelona, Catalunya E08003, Spain; Department of Marine Biology and Oceanography, Institut de Ciències del Mar, CSIC. Pg Marítim de la Barceloneta 37-49, Barcelona, Catalunya E08003, Spain; Instituto de Oceanografía y Cambio Global, Universidad de Las Palmas de Gran Canaria, Parque Científico Tecnológico Marino de Taliarte, s/n, Telde, Las Palmas 35214, Spain; Department of Marine Biology and Oceanography, Institut de Ciències del Mar, CSIC. Pg Marítim de la Barceloneta 37-49, Barcelona, Catalunya E08003, Spain; Department of Marine Biology and Oceanography, Institut de Ciències del Mar, CSIC. Pg Marítim de la Barceloneta 37-49, Barcelona, Catalunya E08003, Spain; Red Sea Research Centre (RSRC), King Abdullah University of Science and Technology, Thuwal 23955, Saudi Arabia; Department of Marine Biology and Oceanography, Institut de Ciències del Mar, CSIC. Pg Marítim de la Barceloneta 37-49, Barcelona, Catalunya E08003, Spain

**Keywords:** global ocean, microbial communities, community dynamics, metabarcoding, RNA, DNA

## Abstract

A persistent microbial seed bank is postulated to sustain the marine biosphere, and recent findings show that prokaryotic taxa present in the ocean’s surface dominate prokaryotic communities throughout the water column. Yet, environmental conditions exert a tight control on the activity of prokaryotes, and drastic changes in these conditions are known to occur from the surface to deep waters. The simultaneous characterization of the total (DNA) and active (i.e. with potential for protein synthesis, RNA) free-living communities in 13 stations distributed across the tropical and subtropical global ocean allowed us to assess their change in structure and diversity along the water column. We observed that active communities were surprisingly more similar along the vertical gradient than total communities. Looking at the vertical connectivity of the active vs. the total communities, we found that taxa detected in the surface sometimes accounted for more than 75% of the active microbiome of bathypelagic waters (50% on average). These active taxa were generally rare in the surface, representing a small fraction of all the surface taxa. Our findings show that the drastic vertical change in environmental conditions leads to the inactivation and disappearance of a large proportion of surface taxa, but some surface-rare taxa remain active (or with potential for protein synthesis) and dominate the bathypelagic active microbiome.

## Introduction

Microbes drive marine biogeochemical cycles, dominating the cell abundance, diversity, and metabolic activity of the ocean. High throughput sequencing techniques have revolutionized the field of microbial ecology, shedding light onto the vast ocean prokaryotic diversity [1] and the existence of a “rare biosphere” (i.e. low abundant taxa [2]) that act as reservoir for most phylogenetic and functional diversity [3].

Global-scale expeditions conducting microbial metabarcoding and metagenomics have provided evidence of biogeographic patterns in both the horizontal and vertical dimension [1, ^4–6^]. The drastic change in environmental conditions along the water column is a major driver of ocean microbial communities structure, as evidenced by the marked changes in community composition from the sunlit ocean to the ocean’s interior [1, 7, 8]. However, it has been recently shown that most of the taxa detected at any given depth can also be detected in surface waters [9]. Sinking particles have been proposed as one of the main dispersal vectors of surface taxa [9, 10], seeding deep ocean communities upon detachment [e.g. 11]. Since community composition is mainly governed at ecological timescales by both selection and dispersal [12], it is possible that these dispersed surface taxa become inactive during their transit to the deep ocean interior and do not play a role in bathypelagic metabolism. Indeed, dormant prokaryotes can remain inactive for decades to millennia [^13–16^], and thus persistency weakens the strength of species selection, potentially increasing the apparent similarity between communities [17, 18].

The use of single-cell approaches in the late 1990s and early 2000s already unveiled the presence of sizeable proportion of prokaryotic cells that could be dormant or even dead in marine communities [19]. Despite this, most biogeography and community assembly studies in the ocean have been based on DNA methods, which do not account for the metabolic heterogeneity of prokaryotes, as these methods cannot discriminate among dead, dormant, slow-growing, and fast-growing cells. In the early 2010s, sequencing of the ribosomal RNA started to be widely used to characterize the growing or active microbes within communities, providing interesting insights into spatial and temporal dynamics of potentially active bacteria and archaea in coastal and open ocean waters [^20–25^]. Yet, the rise of some concerns about the limitations of this approach to delineate active prokaryotes, as thoroughly discussed in References [26, 27], discouraged its use in ocean systems. Nonetheless, despite its limitations, the simultaneous characterization of 16S rRNA transcripts and genes offers the option to delineate the most reactive members of the community, as RNA is indicative of (recent or current) potential for protein synthesis [26]. Indeed, its use in freshwater systems has proven highly informative to unveil microbial community assembly and implications for ecosystem functioning [12, 28, 29].

Here we used a combination of 16S rRNA genes and transcripts sequencing to explore community assembly along the water column (3–4000 m depth) of the global tropical and subtropical ocean. We first compared the diversity and taxonomic composition of free-living total communities (DNA-based) with communities with potential for protein synthesis (RNA-based communities, hereafter “active” communities) and how they changed with depth. Then, we explored the vertical connectivity of total and active communities along the water column, with the aim of assessing (i) the proportion of surface taxa that reach the deep ocean and (ii) whether these surface-dispersed taxa become metabolically inactive in the deep ocean because of negative environmental selection upon encountering drastic changes in environmental conditions.

## Materials and methods

### Sample and environmental data collection

Samples were collected during the Malaspina 2010 Circumnavigation expedition (December 2010 to July 2011) [30]. Thirteen stations distributed across the subtropical and tropical Pacific, Atlantic, and Indian oceans were sampled, covering distinct Longhurst provinces (Fig. S1), at seven depths ranging from the surface to the bathypelagic, representing a total of 91 water samples. Samples were collected with Niskin bottles mounted on a rosette sampler equipped with conductivity–temperature–depth (Seabird SBE 911) and dissolved oxygen (SBE 43) profilers, whereas the surface one was taken with a large oceanographic bottle. Apparent oxygen utilization was calculated as the difference between the saturation and measured dissolved oxygen concentrations. Samples for inorganic nutrients (NO_3_^−^, NO_2_^−^, PO_4_^3−^, and SiO_2_) were kept frozen, and measured spectrophotometrically using an Alliance Evolution II autoanalyzer following Reference [31]. In specific meso- and bathypelagic samples, missing data on nutrient concentrations were taken from the World Ocean Database [32].

### Nucleic acids extraction and sequencing

For nucleic acids, 12-L samples were prefiltered through a 200-μm and a 20-μm meshes to remove large plankton and then sequentially filtered using a peristaltic pump through 142-mm diameter (Isopore, Millipore) 3- and 0.2-μm pore-size polycarbonate filters, as described in Reference [33]. Filtration time ranged between 15 and 20 min. Filters were then flash frozen in liquid nitrogen and stored at −80°C until nucleic acid extraction. The whole process from sample retrieval to filter storage lasted 3–4 h during which the samples were kept in the dark close to their in situ temperature (at 4°C for the deep ocean samples). rRNA is considered stable at this temperature, and depressurization during recovery does not seem to affect the rRNA diversity of deep ocean prokaryotic communities [34].

Once in the laboratory, the 0.2-μm polycarbonate filters were cut into small pieces and cryogrinded with a Freezer-Mill 6770 (Spex) using 3 cycles of 1 min, to maximize cell lysis. RNA and DNA were extracted simultaneously from the same filter using the NucleoSpin RNA kit and the NucleoSpin RNA/DNA buffer set (Macherey-Nagel) following manufacturer’s instructions as described in Reference [33]. Contamination with residual DNA in the RNA extracts was checked by PCR with universal prokaryotic primers and, if detected, was removed using the Turbo DNA-free kit (Applied Biosystems). RNA was reverse transcribed to cDNA using random hexamers and the SuperScriptIII kit (Invitrogen) according to the manufacturer’s instructions. DNA and cDNA were then quantified using a Qubit fluorometer assay (Life Technologies, Paisley, UK). The V4–V5 regions of the 16S gene were amplified with the primers 515F and 926R [35] and sequenced in an Illumina MiSeq platform using 2 × 250 bp paired-end approach at the RTLGenomics facility (Lubbock, Texas, USA; https://rtlgenomics.com/). Illumina reads obtained from DNA and cDNA extracts (DNA and RNA sets, respectively) were processed together using DADA2 [36] after removing primers and spurious sequences using cutadapt [37]. The parameters used for DADA2 were trunclen = (220,200) and maxEE = (2,4), and taxonomic assignment was performed using the “assignTaxonomy” function against SILVA v.138.

To minimize the potential effect of various 16S rRNA copies in the contribution of individual taxa to the total and active communities, the relative abundance of each amplicon sequence variant (ASV) was normalized by their average 16S rRNA gene copy number using the tool “estimate” of the ribosomal RNA copy number database [38]. For the calculation, we took into account the taxonomic resolution available for each ASV and used the estimated value for the lowest available taxonomic rank (order, family, or genus, Table S1). When the lowest available taxonomy rank for the ASVs was Class, we did not correct for 16S rRNA copy number as its inaccurate prediction may introduce larger bias in community composition [39].

### Prokaryotic abundances and bulk heterotrophic activity

Prokaryotic abundance and the proportion of high nucleic acid cells were determined by flow cytometry as described in Reference [40]. Prokaryotic cell volume was estimated using the standardized (to beads) SSC flow cytometry signal assuming a spherical shape, as in Reference [41]. Bulk prokaryotic heterotrophic activity was estimated using ^3^H-leucine incorporation as detailed in Reference [42].

### Data treatment

All data treatment and statistical analyses were conducted with the R Statistical Software using version 4.0.0.

To calculate diversity indexes, the ASV table was rarefied 100 times down to 10 000 reads using the “rrarefy” function in the “vegan” 2.5-7 package. Prokaryotic richness (the number of ASVs per sample) and the Shannon index for each sample were calculated using the “vegan” 2.5-7 package for the individual permutations and then the resulting 100 values were averaged. Sample evenness was calculated using the Pielou index (*J* = *H*/ln(nASV), where *H* is the Shannon index, and nASV the richness of every sample.

The phylogenetic diversity (or Faith’s index [43]) for each sample was calculated considering the evolutionary relationships among ASVs using the computed phylogeny, as the sum of the lengths of all the branches in the phylogeny. For this, we used the “decipher” 2.16.1 [44] and “phangorn” 2.5.5 [45] packages. Differences between DNA and RNA for richness/diversity measures were tested using Mann–Whitney tests, as data normality was not assured. Statistical differences among the different depth layers were tested using Kruskal–Wallis and Dunn post hoc tests (performed using the “fsa” package version 0.9.3). Rarefied abundance tables (mean abundance after 100 permutations) were used for all the diversity analyses, the taxa recruitment analyses, and the taxonomic composition of the communities.

Nonmetric Multidimensional Scaling (NMDS) was performed using the “vegan” package [46] using Euclidean distances of centered-log-ratio (CLR) transformed DNA and RNA non-rarefied ASV abundance tables [47]. This transformation was performed in order to reduce compositional artifacts [47]. Permutational multivariate analysis of variance (PERMANOVA) was conducted with 1000 permutations to test for significant differences between groups of samples using molecule (DNA or RNA) and depth layer as the grouping variables.

To calculate ribosomal RNA:DNA ratios, a pseudo-count of 1 was added to the RNA and DNA data sets. This allowed the calculation of log2 transformed ratios for those ASVs that are inactive (RNA = 0) in some of the samples. Ratios were calculated only for those phyla representing more than 500 reads in the DNA data set.

To assess the vertical connectivity of ASVs, we followed a similar approach as in Reference [9], categorizing all ASVs based on the first depth they were detected assuming a directionality from the surface (d1) to the bathypelagic (d7). To analyze which ASVs showed the largest changes in RNA reads abundance, we calculated the average Euclidean distance of the RNA reads abundance of each ASV between all samples, using the R-script presented in Appendix S1 of Reference [48]. Those ASVs showing a mean distance > 10 were considered “shifters.” This value represented an average maximum change of 611 reads between two samples for any shifter (range 43–6507). We then categorized the shifters based on their depth preference: if their mean RNA abundance was higher in surface waters than in the bathypelagic we classified them as “sunlit” shifters, whereas if their mean RNA abundance was higher in the bathypelagic than in surface waters, we classified them as “deep” shifters. Afterward we extracted those shifters that had been categorized as d1 (detected in the surface) and explored their changes in the contribution to the RNA communities and their RNA:DNA ratio.

## Results

### Vertical structure of total and active prokaryotic communities

**Figure 1:**
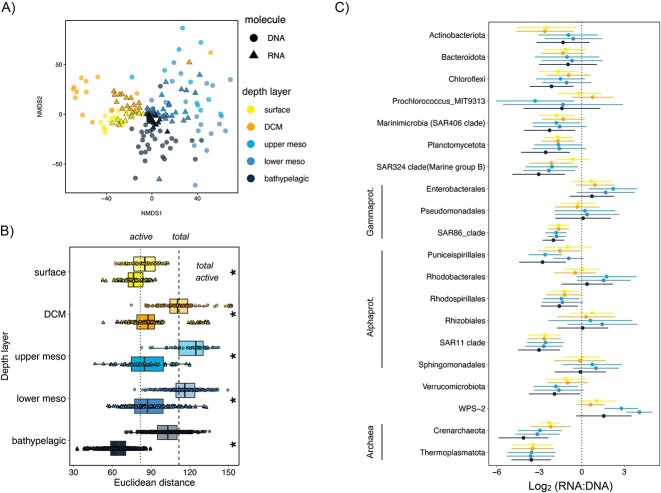
Similarity of total (DNA) and active (RNA) prokaryotic communities and RNA:DNA ratios throughout the water column across the global tropical and subtropical ocean. (A) NMDS ordination of the DNA- (dots) and RNA (triangles)-based communities using Euclidean distances of CLR transformed abundances. Symbols are colored based on the depth layer the samples belong to. (B) Box-plots of Euclidean distances between samples within each depth layer. Dashed line represents the mean distance between DNA samples (dots), whereas the dotted line represents the mean distance between RNA samples (triangles). Significant differences between DNA and RNA communities are labeled with an asterisk (Kruskal–Wallis and post hoc Dunn tests *P* < .01). Values above the mean indicate communities more different than the mean difference between pairs of samples, and values below the mean indicate communities less different than the mean difference between pairs of samples. (C) Log2 (RNA + 1):(DNA + 1) ratios of phyla that represented more than 500 reads in the DNA pool. The taxonomic groups are divided at the phylum level except for proteobacteria that are at the class level, and Alphaproteobacteria and Gammaproteobacteria that are at the order level.

The tropical and subtropical stations sampled spanned waters with a broad range of environmental conditions (Fig. S2). The NMDS ordination of total (DNA) and active (RNA) communities showed a clear segregation of the samples based on depth, as supported by PERMANOVA analysis (Table 1) for both the total and active communities, considered either separately or combined. These differences were particularly evident between epipelagic (surface and Deep Chlorophyll maximum—DCM) and dark ocean samples (Fig. 1A), likely following the changes in environmental conditions (Fig. S2). Total communities diverged from the active portion of these communities throughout the water column (Fig. 1A, Table 1), whereas active communities were more similar than total communities within each depth (i.e. lower between-sample Euclidean distances, Kruskal–Wallis and post hoc Dunn tests *P* < .01) (Fig. 1B). Surface total communities showed lower dispersion (i.e. lower Euclidean distances) than the global mean difference between pairs of samples (dashed line in Fig. 1B), suggesting they differed little across the different biomes sampled compared with the rest of the depth layers considered (Fig. 1B). In contrast, the active communities presented the lowest dispersion in the bathypelagic, indicating that the active fraction of the communities changed less from sample to sample in the bathypelagic than in the rest of the depth layers considered. The highest heterogeneity in composition between communities was found for the upper mesopelagic (200–500-m depth) in the case of total communities (highest Euclidean distances, Fig. 1B), and in the DCM and lower mesopelagic (500–1000 m) for the active fraction.

**Table 1:** PERMANOVA was used to evaluate if the depth layer exerted a significant role in the structuring (or grouping) of the communities. Results are based on total (DNA) and active (RNA) communities Euclidean distance matrices considered either independently or together.

**Molecule**	**Group**	**df**	**F-statistic**	***R*** ^**2**^	***P*-value**
DNA	Depth layer	4	6.31	0.227	<.001
RNA	Depth layer	4	1.391	0.060	.0069
DNA and RNA	Depth layer	4	10.396	0.187	<.001
	molecule	1	5.499	0.025	<.001

The distinct patterns observed in the structure of total and active communities were also reflected in the taxonomic composition: both surface active and total communities had a large contribution of *Prochlorococcus* throughout the global tropical and subtropical ocean (Fig. S3), explaining the community similarity seen in Fig. 1, whereas aphotic total and active communities markedly differed in the dominant taxonomic groups, with the total communities having a notable contribution of the SAR11 clade and Crenarchaeota that was negligible in the active communities. In contrast, the active communities were largely dominated by other groups, such as Enterobacterales (mainly *Alteromonas*), Rhodobacterales, Sphingomonadales, and Pseudomonadales. These trends were further explored by looking at the RNA:DNA ratios of the different taxonomic groups in the different depth layers sampled. Groups like the SAR86 clade (gammaproteobacteria), SAR11 clade (alphaproteobacteria), SAR324 clade and the archaeal phyla Crenarchaeota, and Thermoplasmatota, contributed more to the community DNA pool than to the RNA pool, whereas groups like the gammaproteobacterial Enterobacterales, and the candidate phylum WPS-2, were always overrepresented in the RNA pool (Fig. 1C). Some depth-related trends could also be detected in the RNA:DNA relationships of the different groups, such as a larger metabolic activation of some groups in the mesopelagic layers (see e.g. Enterobacterales, Rhodobacterales, Rhizobiales, Sphingomonadales, and WPS-2, in Fig. 1C), and the expected low RNA:DNA ratio of Prochlorococcus in the aphotic ocean.

The most evident biogeographic pattern across ocean basins was that members of the Actinobacteriota were mostly absent from bathypelagic communities in the Atlantic, but represented a large fraction of total and active communities in the Pacific. In contrast, Enterobacterales were more abundant in Atlantic and Indian Ocean waters, massively dominating some of the active dark ocean communities at some stations (Fig. S3). Noticeably, some stations that belonged to different ocean basins but were close in distance and likely connected through thermohaline circulation, such as Stations 39 and 49 (Fig. S1), showed very similar patterns of taxonomic distribution in the total communities, but very different patterns in the active fraction of the community (Fig. S3).

### Patterns of diversity

**Figure 2:**
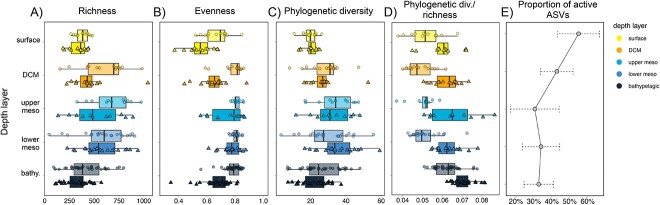
Changes in diversity indexes in the total and active communities, and in the proportion of active taxa throughout the water column. Box-plots of (A) richness, (B) evenness, (C) phylogenetic diversity, and (D) the ratio of phylogenetic diversity/richness within each depth layer for the total (DNA, upper boxplot, dots) and active (RNA, lower boxplot, triangles) communities. (E) Line plot of the proportion of active taxa (mean + SD) along the water column.

Taxonomic richness was higher for the total communities than for the active ones at all depths (Mann–Whitney test, *P* = .005), supporting the view that only a portion of the community is made of metabolically active cells (Fig. 2A). Both total and active communities displayed maximum richness in the mesopelagic. Community evenness showed a similar pattern to richness for the active communities, with maximum values in the mesopelagic, but it was rather uniform with depth in the total communities except for the surface ones that showed comparatively more uneven distributions. Overall, total communities displayed higher evenness than the active ones (Mann–Whitney test, *P* < .001) (Fig. 2B). We also computed the phylogenetic diversity of the samples, to obtain a measure of the phylogenetic breadth of the total and active prokaryotic communities. This phylogenetic diversity varied along the water column following the patterns of richness (Fig. 2C), although the differences between total and active communities were not significant (Mann–Whitney test, *P* = .77). The ratio between phylogenetic diversity and richness increased slightly with depth for both the active and total communities (Fig. 2D), and was higher in the active communities throughout the water column (Mann–Whitney test, *P* < .001), indicating that the active communities, despite being globally less diverse than the total communities, were composed by more phylogenetically divergent phylotypes.

The relative proportion of active taxa (detected in both the DNA and RNA pools) decreased with depth (Fig. 2E), showing lower values in the aphotic ocean.

### Vertical connectivity in total and active prokaryotic communities throughout the ocean depth layers

Vertical connectivity was assessed following the same approach as in Reference [9], analyzing the proportion of taxa (ASVs) recruited at the different sampled depths assuming directionality from the surface to the deep ocean. The reason behind this assumption is that sinking particles are considered one of the main dispersion mechanisms of prokaryotes in the ocean, seeding dark ocean free-living communities when prokaryotes detach from particles [9, 49]. We categorized all ASVs into seven depth groups (from d1, surface, to d7, bathypelagic), defined by the depth at which they were first detected at each of the stations. Hence, ASVs detected in the surface (d1) of a given station were categorized as d1 ASVs, whereas ASVs that were first detected at the following depth in that same station were categorized as d2 ASVs. We then evaluated the contribution of all these categories to total and active communities at each of the seven depths in each of the stations.

**Figure 3:**
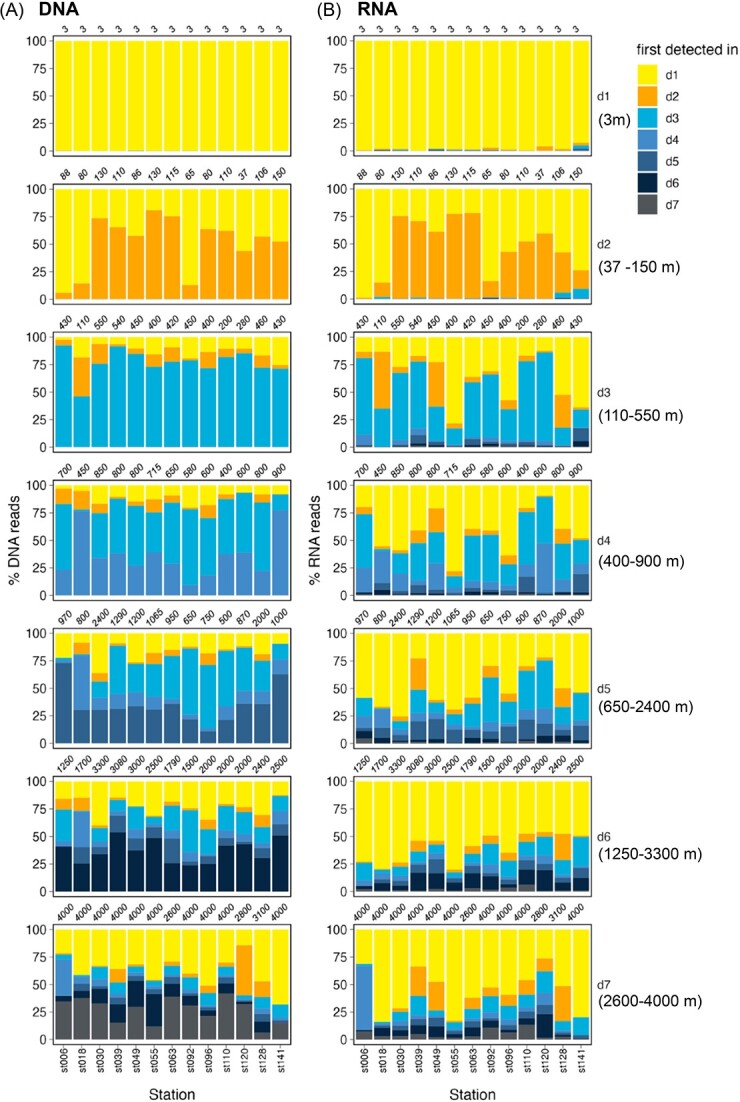
Taxa detected at the surface dominate active (RNA) communities in the bathypelagic. Contribution of ASVs categorized as the first depth they were detected in the DNA pool to (A) DNA and (B) RNA communities at the different depth layers (assuming a directionality from surface to the bathypelagic). The labels on top of each graph represent the depths from which samples were collected at each of the stations. The ranges of depths considered at each depth levels are shown on the right; d1 = 3 m, surface; d2 = 37–150 m (median 106 m), ~DCM; d3 = 110–550 m (median 430 m), d4 = 400–900 m (median 700 m), d5 = 650–2400 m (median 970 m), d6 = 1250–3300 m (median 2000 m), d7 = 2600–4000 m (median 4000 m). Note that in the surface RNA communities (panel b, d1), there are some taxa with a different category than d1 because of the presence of phantom taxa, which are those present at such low abundances that are undetected in the DNA pool but active enough to be detected in the RNA pool.

There was a notable recruitment of new ASVs to the total communities when moving from one depth to the following one, particularly in the DCM (d2) and upper mesopelagic (d3, <500 m depth), where the recruited ASVs represented up to 75% and ~90% of the community sequences of the DNA pool, respectively (Fig. 3A). In deeper layers, newly recruited ASVs accounted for a lower proportion of the community sequences, generally between 25% and 30%, albeit with some exceptions. The deepest samples (d7) had comparatively higher contributions of surface ASVs sequences than the previous depths, representing, on average, 50% of bathypelagic communities. This agrees with previous studies showing a higher connectivity between surface and bathypelagic samples, than between surface and intermediate waters [10, 50], likely driven by fast sinking particles with short residence times within the mesopelagic, and possibly active transport by diel vertical migrants [51].

Surface-derived ASVs generally dominated the active communities throughout the water column (Fig. 3B), except at the DCM and upper mesopelagic (d3), where taxa recruited within these layers accounted for more than 50% of the active community in half of the stations. Taxa detected at the surface accounted on average for 50% of the active microbiome in the deepest samples.

**Figure 4:**
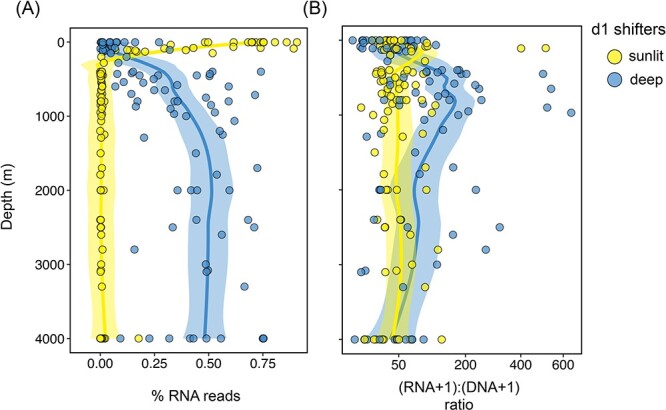
Exploration of those d1 ASVs that changed most drastically in their RNA abundances from the surface to the bathypelagic (d1 shifters). (A) Summed (per-sample) contribution to RNA reads, (B) mean per-sample (RNA + 1):(DNA + 1) ratio of the “sunlit” shifters (those d1 ASVs showing higher RNA abundances in the surface than in the bathypelagic) and the “deep” shifters (those d1 ASVs showing higher abundances in the bathypelagic than in the surface). The solid lines represents a best fit smooth curve through the center of the data calculated using weighted least squares to highlight the trends, the shaded area represent the confidence interval around the smooth.

We further investigated those surface (d1) ASVs that changed most drastically in their contribution to the RNA pool between the surface and the bathypelagic layer (here after referred as d1 shifters, see Materials and Methods for further details). These shifting ASVs were categorized as “sunlit” if they displayed higher RNA abundances in the surface layer than in the bathypelagic, and “deep” if they displayed higher RNA abundances in the bathypelagic than in the surface. We found that “sunlit” d1 shifters decrease in RNA abundance sharply below the photic ocean (Fig. 4A), whereas “deep” d1 shifters were present throughout the water column, increasing their RNA abundances toward the lower mesopelagic and remaining rather constant from 1000 to 4000 m (Fig. 4A). Looking at the variations in RNA:DNA ratio of these shifters, we found that the “sunlit” shifters had comparatively higher ratios in the photic layer than in the aphotic ocean, but ratio values were generally low and rather uniform with depth (Fig. 4B). In contrast, “deep” shifters displayed marked maximum ratios in the lower mesopelagic (500–1000-m depth), and these ratios decreased steadily toward the deep ocean.

**Figure 5:**
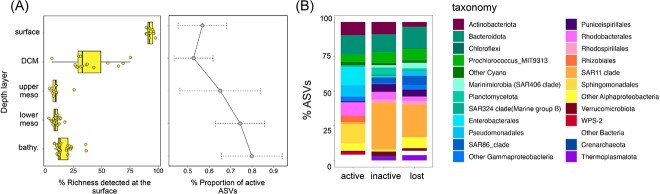
Only a small fraction of surface taxa reach the bathypelagic. (A) Box plot showing the change in richness of the surface derived taxa at the different depth layers in the DNA communities. The line plot indicates the proportion of active ASVs (mean + SD) within that pool of surface taxa at each depth layer. (B) Taxonomic affiliation of surface-derived ASVs that are active, inactive, or have been lost in the bathypelagic samples.

We then explored the shifts in the richness of the pool of surface (d1) taxa detected at the different depths, and found a marked decrease in richness from the surface to the aphotic layers. Only ~15% of the total surface taxa could be detected at the deepest layers (Fig. 5A). This implies that species sorting results in only a subset of the taxa detected at the surface being able to thrive when dispersed toward the ocean’s interior, but these few taxa are responsible for a large proportion of the active microbiome (Figs 3B and 4A). Looking at the proportion of active taxa in this pool of surface taxa along the water column, we found that deeper layers had a larger proportion of active taxa in comparison to surface waters (Fig. 5A, right panel), suggesting that surface taxa that become inactive end up being lost or are diluted below the detection limit of our sequencing approach. We then analyzed the taxonomic distribution of (i) the surface ASVs that were undetected (i.e. “lost”) in the deepest samples (2600–4000 m), (ii) surface taxa that were detected in these samples but inactive, and (iii) taxa that remained active. The taxonomic compositions of undetected and inactive taxa were strikingly similar (Fig. 5B), supporting our hypothesis that inactive taxa end up dying or falling below our detection limit. Among the inactive or undetected taxa we found a large proportion of ASVs from typical surface oligotrophs, such as SAR11, SAR86, and Puniceispirillales (SAR116). *Prochlorococcus* ASVs also appeared in the inactive/undetected categories, although some ASVs were detected in the RNA pool, likely reflecting recent arrivals accompanying fast-sinking particles [11, 52, 53]. In contrast, the contribution of Enterobacterales, Pseudomonadales, Rhizobiales, and Sphingomonadales ASVs largely increased in the active fraction of the communities.

**Figure 6:**
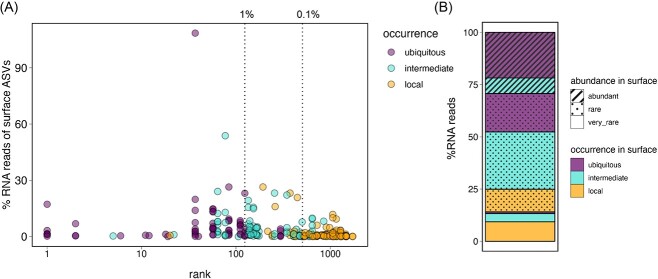
Abundant and rare surface-derived taxa contribute to the bathypelagic active microbiome. (A) Rank abundance distribution of ASVs in the bathypelagic (d7) ordered by their total abundance at the surface (Rank 1 is the most abundant). Dotted vertical lines represent the standard divisions between abundant and rare (1%) and very rare (0.1%) ASVs. Color code represents the occurrence of the ASVs in the surface samples: ubiquitous (>70% of the stations), intermediate (30%–70% of the stations), and local (<30% of the stations). (B) Percent contribution of the different categories to the surface-derived taxa that are active in the bathypelagic.

We next investigated whether the surface taxa that dominate the active communities in the bathypelagic were abundant (>1% of relative abundance in at least one of the surface samples), rare (0.1%–1% of relative abundance in at least one sample), or very rare (<0.1% of relative abundance) in the surface DNA communities. The taxa were also categorized based on their occurrence as ubiquitous (present in >70% of stations), intermediate (present in 30%–70% of stations), or local (present in <30% of stations). Whereas active surface communities were overall dominated by abundant and ubiquitous taxa (Fig. S4A), surface rare and very rare ASVs accounted for notable fractions of the active bathypelagic communities (Figs 6A and S4), representing around 70% of the surface-derived RNA pool (Fig. 6B). Some rare taxa were ubiquitous, but others had a local or intermediate distribution. In contrast, the very rare surface taxa that became abundant in the bathypelagic active communities (>1% relative abundance in the RNA pool in at least one sample) generally had a local distribution in the surface. We further checked if the surface-derived taxa that were abundant in the bathypelagic active microbiome were cosmopolitan throughout the water column, and found that 94% of them were found at the seven depths sampled and the remaining 6% were detected at five out of the seven depths (details not shown). This may imply that water-column cosmopolitan taxa play an important role in the bathypelagic microbiome.

Among the abundant and ubiquitous surface-derived taxa, we found some members of the alphaproteobacterial order Rhodobacterales and Sphingomonadales, Actinobacteriota, and some *Prochlorococcus* (Fig. S5), although the presence of the later likely reflects recent sinking events. Among the rare taxa, we found the alphaproteobacterial Rhizobiales, and the gammaproteobacteria Enterobacterales (mainly *Alteromonas*). Surface very rare taxa that were active in the bathypelagic belonged mostly to the Rhizobiales, and the candidate phylum WPS-2.

## Discussion

### Global patterns in diversity of total and active communities

Here we analyzed the total and active (i.e. with potential for protein synthesis) communities in the global tropical and subtropical ocean from the surface to the bathypelagic layer (~4000 m). Active communities differed largely from total communities throughout the water column, as observed in other ecosystems [54, 55]. Despite the marked environmental gradients encountered with depth, the active communities were comparatively more similar among them than the total communities were (Fig. 1A). This means that a large fraction of the RNA pool is dominated by similar taxa regardless of water depth.

A general trend observed was that the active populations in the aphotic layer of the Atlantic and Indian Ocean were often overwhelmingly dominated by a few taxonomic groups, such as Enterobacterales (mainly *Alteromonas*) and Rhodobacterales, whereas in the Pacific Ocean Actinobacteriota had a notable contribution (Fig. S3). This may be related to the characteristics of the dissolved organic matter pool in the dark Pacific Ocean, which is ~2000 years older [56], and thus likely more recalcitrant, and has lower concentrations than in the Atlantic and the Indian Oceans [57]. The presence of Actinobacteriota in these aged waters agrees with the view that this phylum can utilize a broad spectrum of refractory compounds [58].

The RNA:DNA ratios showed in general two contrasting trends, with some groups being overrepresented in the RNA (i.e. log_2_ ratios higher than 0) throughout the water column and some groups overrepresented in the DNA (Fig. 1C). Among those overrepresented in the DNA were typical oligotrophs like the SAR11 and SAR86 clades, which have been reported to display low RNA:DNA ratios [20, 24, 59], slow growth rates [^60–63^], and metabolic streamlining [64, 65]. Other slow growers like Crenarchaeota [66] were also overrepresented in the DNA, together with Marinimicrobia (SAR402), Planctomycetota, and Verrucomicrobiota. In contrast, groups such as Enterobacterales and WPS-2, throughout the water column, or Pseudomonadales, Rhodobacterales, Sphingomonadales, and Rhizobiales, in the dark ocean, were overrepresented in the RNA pool (Fig. 1C). Enterobacterales and Rhodobacterales have been reported to display high RNA:DNA ratios [59] and fast growth rates in the ocean and estuaries [61, 62, 67], in agreement with the view that high RNA:DNA ratios are indicative of high metabolic activity. Despite RNA:DNA ratios are subject to several sources of variation, such as translation efficiency [68], or changes in the growth phase or physiological status [26, 60], they have indeed been successfully used to infer growth rates of marine prokaryotes [25, 59, 60]. We thus posit that the water column trends observed in the RNA:DNA ratios in the tropical and subtropical ocean provide information about the metabolic strategies of different marine prokaryotic groups.

The richness of both total and active communities presented a maximum in the mesopelagic layer, consistent with the postulated role of the mesopelagic layer as a hotspot of microbial activity and diversity [33, 69, 70]. The richness of the total communities was higher than that of the active communities, which agrees with the existence of a dormant seedbank that may be activated upon changes in environmental conditions [13]. Nonetheless, despite showing overall lower richness (Fig. 2A), active communities were more phylogenetically diverse (Fig. 2D). This implies less micro-diversity within the active populations, perhaps to avoid functional redundancy. The proportion of active taxa decreased toward the ocean’s interior in accordance with resource limitation in the dark ocean (Fig. 2E).

### The dark ocean active prokaryotic microbiome is dominated by a few surface taxa

As surface-derived sinking particles are considered one of the main vectors of microbial dispersal along the water column, we explored the connectivity of communities from the surface toward the ocean’s interior (Fig. 3). This analysis demonstrated that taxa already detected in the surface represented a large fraction of the deep ocean total communities, as shown previously in a different sampling [9]. The contribution of surface ASVs to free-living deep ocean total communities was lower here than in that study, probably because they used lower taxonomic resolution (97% OTUs instead of ASVs) and categorized as surface OTUs those detected at any station in the surface, whereas our analysis was done independently for each of the stations. In fact, that previous study [9] found lower contribution of surface OTUs to deep ocean communities when each station was analyzed separately (Fig. S6 of Reference [9]). Nonetheless, in both studies, the contribution of surface detected taxa increased in bathypelagic waters compared with intermediate waters (Fig. 3), supporting the current view that there is a strong connectivity between surface and bathypelagic communities driven by fast-sinking particles [10, 50].

Regarding the active communities, surface ASVs dominated deep ocean communities, representing up to 90% of the RNA reads (Fig. 3B). Yet, the pool of surface-derived taxa that reached deep waters was only a small fraction (~15% on average, Fig. 5) of the taxa detected in the surface, whereas the majority of surface taxa became lost or undetected in deep waters (Fig. 5). This agrees with our hypothesis that most surface-derived taxa become inactive as they are transported to deeper waters because of negative environmental selection (exemplified by the sharp decrease in the contribution to the RNA pool of the “sunlit shifters” in Fig. 4, and the marked decrease in richness in Fig. 5A), but also indicates that a few surface taxa are able to prevail throughout the water column. Given the poor taxonomic resolution of the 16S rRNA gene, it is possible that different ecotypes are hidden within these cosmopolitan ASVs [71]. Yet, despite our analysis focused on the free-living component of the community (0.2–3-μm size fraction, [^72–74^]), most of the surface-derived taxonomic groups that were active in the bathypelagic have been described to have a preference for a particle-associated lifestyle [75, 76]. Groups like *Alteromonas* (Enterobacterales) and Rhodobacterales, which accounted for a large proportion of the RNA reads, have been found in sinking particles arriving to the bathypelagic in periods of elevated carbon flux [77], and are also widely distributed along the water column either in the free-living or the particle associated size fraction [49, 75, 78, 79], probably using the particles as dispersal vectors [9, 50, 77]. The fact that surface-associated genes are often found in deep ocean metagenome-assembled genomes of the particle associated fraction [80], and that deep ocean bacterial isolates match at 100% identity 16S gene sequences of free-living and particle-associated bacteria throughout the water column [49], supports this view. The life cycles of bacteria growing on particles are complex, with continuous cycles of attachment and detachment following particle transformation through the action of the particle colonizers [^81–84^]. Thus, sinking particles likely seed free-living deep ocean communities with their associated prokaryotes during these cycles of detachment. Indeed, it was recently reported that particle-associated and dual-lifestyle prokaryotes (those able to thrive both in the particle associated and the free-living realm) may represent ~50% and 75% of bathypelagic free-living total and active communities, respectively [85]. Nonetheless, it is also possible that some of the cells collected in the free-living fraction got detached from the particles during the sampling and filtration processes [86].

Water mass circulation can be another important mechanism for prokaryotic dispersal in the ocean, particularly in deep waters [6, 79], and thus the assumption that vertical connectivity is the strongest mechanism connecting different parts of the ocean could be subject of debate. However, the horizontal dimension explored here is too broad to evaluate any horizontal dispersal patterns and deep water masses circulation occur at much lower speeds (in the order of cm per s-1, [87]) than vertical particle sinking. Moreover, the distribution of groups such as *Alteromonas*, and Rhodobacterales, which dominated the active communities, has recently been shown to be poorly explained by water mass circulation and mixing [79], supporting our vertical connectivity approach.

Although the amount of ribosomes (and therefore RNA sequences) does not always scale linearly with metabolic activity [26], changes in the number of ribosomes are expected to occur upon changes in growth or metabolic rates. For example, despite some gammaproteobacterial isolates, such as *Vibrio* and *Alteromonas*, have been shown to keep intact ribosomes during starvation in pure culture [88], the amount of ribosomes usually decreases dramatically within the first days of starvation [89]. In a long-term starvation experiment with bathypelagic communities in the absence of external organic carbon input, we observed that the contribution of *Alteromonas* to the RNA pool was stable during the first 2 weeks, but then decreased drastically afterward (Fig. S6). Assuming that particles are the dispersal vectors for these taxa, and assuming sinking rates of 200 m d^−1^, at the high end for open ocean waters [90, 91], surface particles would require 20 days to reach 4000 m depth. Considering that particle-sedimentation has an episodic nature [92], including fast-sinking events [53], it is thus unlikely that the dominance of surface taxa in the bathypelagic rRNA communities reflects past activities, because if cells had become inactive during their transit to the deep ocean, their contribution to the RNA pool would be much lower.

The increase in hydrostatic pressure could affect the metabolism of surface-derived prokaryotic taxa [^93–95^], but it was recently shown that >80% of the deep sea prokaryotes are piezotolerant (i.e. they have the same activity level under in situ pressure conditions and depressurized conditions [96]). However, these authors also showed that Alteromonadales, which dominated some active bathypelagic communities in our Atlantic samples, were generally piezosensitive, displaying lower per cell activity under high in situ pressure conditions [96]. Nevertheless, despite this decrease in per cell activity, the potentially surface-derived Alteromonadales were still active in the bathypelagic, as they invested energy in the expression of proteins that were not expressed in shallower layers, such as genes involved in flagellum synthesis [96] that allow them to colonize particles. This implies that despite the negative effect of pressure on their activity, piezosensitive prokaryotes transported via sedimenting particles may contribute to bathypelagic metabolism.

Our work shows that a large fraction of the taxa detected in the surface deactivate and/or disappear in the transit to the ocean interior, but some surface-rare taxa remain active (or with potential for protein synthesis) and may even dominate the bathypelagic active microbiome. This result seems counterintuitive, as environmental conditions, characterized by actual substrate limitation in the deep ocean [97], are thought to exert a tight control on the activity or prokaryotes. Studies using next-generation physiological approaches [98] will shed further light on the role of these taxa in bathypelagic metabolism.

## Supplementary Material

Sebastian_etal_ismecomm_suppInfo_revised_2review_ycae015

TableS1_revised_ycae015

## Data Availability

Sequences will be available at ENA under accession number PRJEB45015**.** Metadata associated to these sequences are available in supplementary Table 1 of Reference [33].
