# Supplementary material for: The active free-living bathypelagic microbiome is largely dominated by rare surface taxa
Source: ISME Commun. 2024 Jan 23;4(1):ycae015. doi: 10.1093/ismeco/ycae015 (PMC10919342; doi:10.1093/ismeco/ycae015)
Supplement: Sebastian_etal_ismecomm_suppInfo_revised_2review_ycae015 [file sebastian_etal_ismecomm_suppinfo_revised_2review_ycae015.pdf]

Supplementary Information for

The active free-living bathypelagic microbiome is largely dominated by rare surface taxa

Marta Sebastián[1*], Caterina R. Giner[1], Vanessa Balagué[1], Markel Gómez-Letona[2], Ramon Massana[1], Ramiro Logares[1], Carlos M. Duarte[3], and Josep M. Gasol[1]

This file contains:

6 supplementary Figures

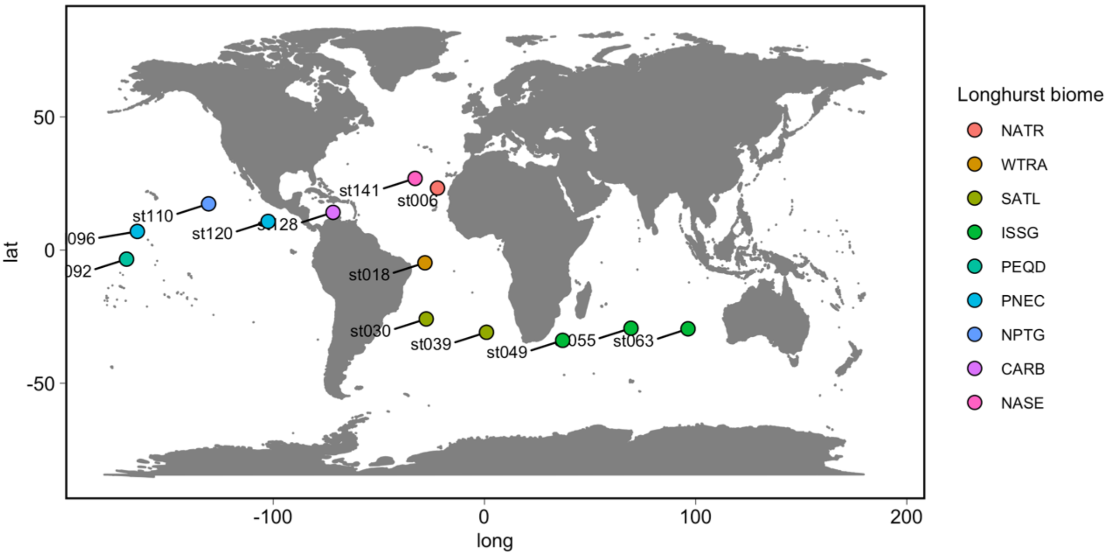

Figure S1. World map showing the location of the sampled stations. Dots are color-coded based on the Longhurst province the samples belong to. NATR: Noth Atlantic Tropical Gyre, WTRA: Western tropical Atlantic, SATL: South Atlantic gyral, ISSG: Indian South subtropical gyre, PEQD: Pacific equatorial divergence, PNEC: North Pacific equatorial counter current, NPTG: North Pacific Tropical gyre, CARB: Caribbean, NASE: Northeast Atlantic subtropical gyral.

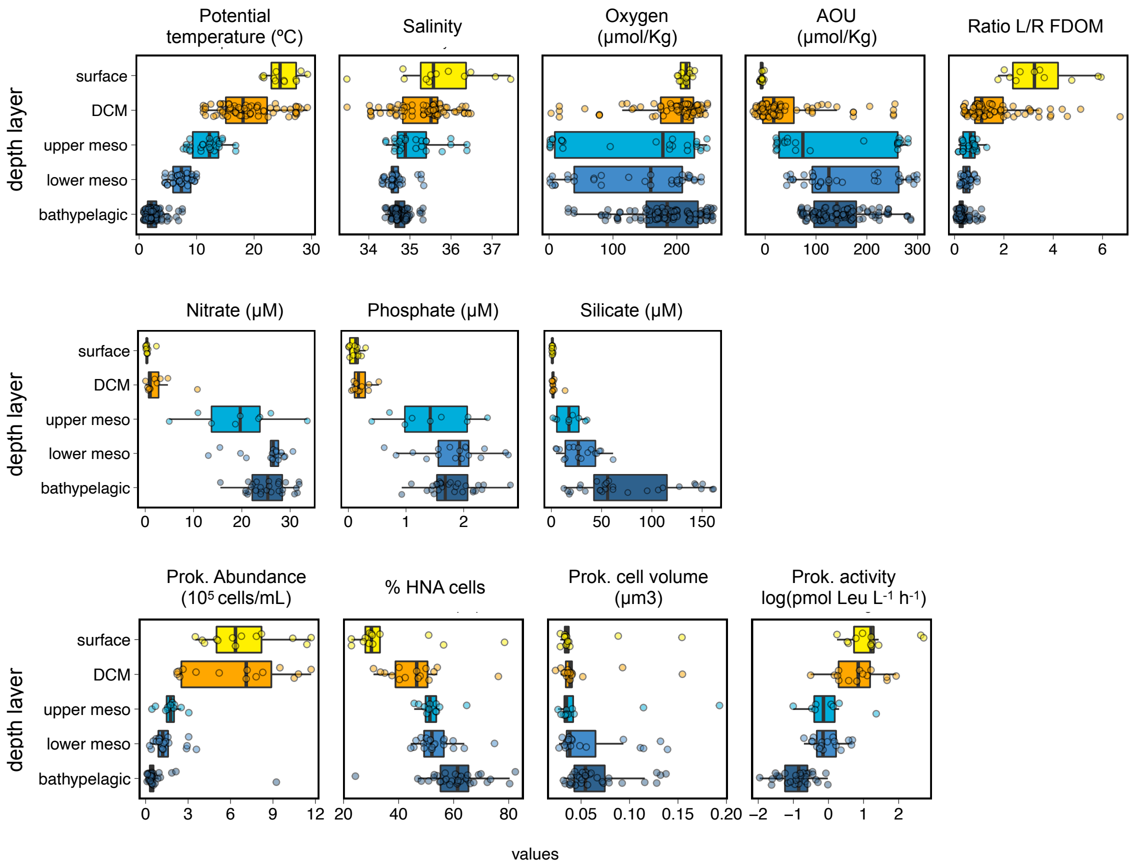

Figure S2. Boxplots showing the range of variation in environmental and prokaryotic variables in the different depth layers considered. AOU: Apparent oxygen utilization, which is a proxy for water ageing. Ratio L/R FDOM: relationship between the labil and recalcitrant components of the fluorescent organic matter, which provides an idea of the quality of organic resources available to bacteria

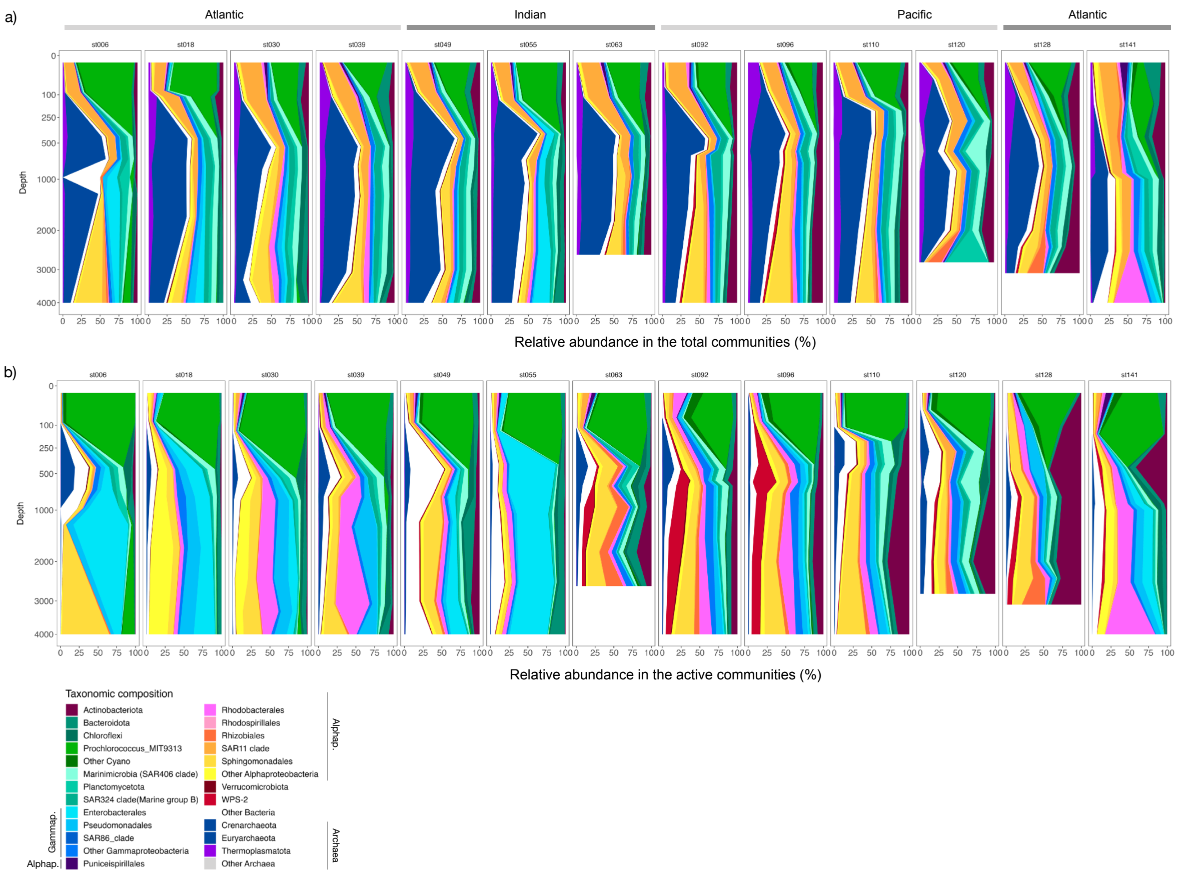

Figure S3. Vertical changes in the taxonomic composition of the a) total and the b) active communities in the different stations sampled. The taxonomic assignation has been performed at the phylum levels except for Alphaproteobacteria and Gammaproteobacteria that are at the order level, and cyanobacteria that are at the genus level. The vertical scale is square-root transformed

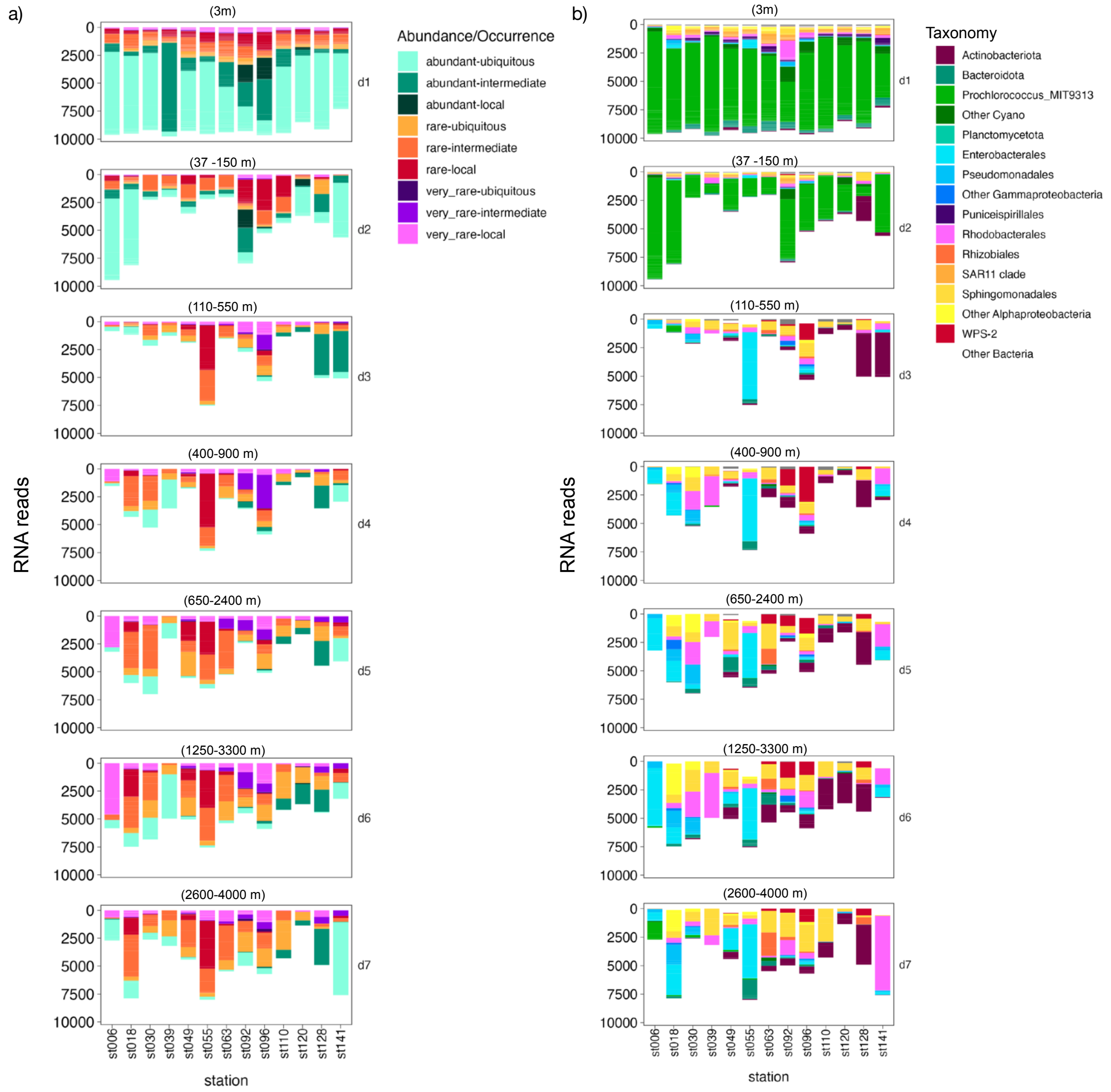

Figure S4. Vertical contribution of the surface taxa to the RNA pool. a) Taxa categorized based on their abundance (abundant: >1% reads, rare between 0.1 and 1% reads and very rare: <0.1% of the reads) and their occurrence (ubiquitous: >70% of the samples, intermediate: between 30 and 70% of the samples, local: <30% of the samples) in the surface. b) Taxonomic assignation of the different taxa at the phylum levels except for Alphaproteobacteria and Gammaproteobacteria that are at the order level, and cyanobacteria that are at the genus level.

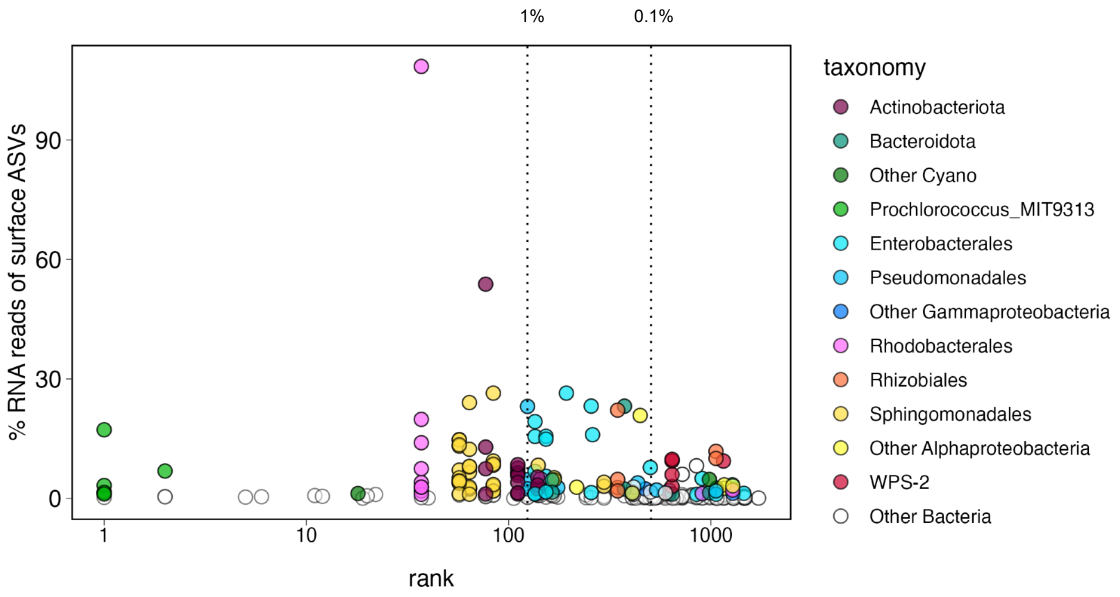

Figure S5. Rank abundance distribution of amplicon sequence variants (ASVs) ordered by their total abundance at the surface (same data as in Fig. 6 of main text). Dotted vertical lines represent the standard divisions between abundant and rare (1%) and very rare (0.1%) ASVs. The x-axis is presented in log-scale to facilitate visualization. Color code indicate the taxonomic affiliation of the ASVs at the phylum levels except for Alpha- and Gammaproteobacteria that are at the order level, or Cyanobacteria that are presented at the genus level.

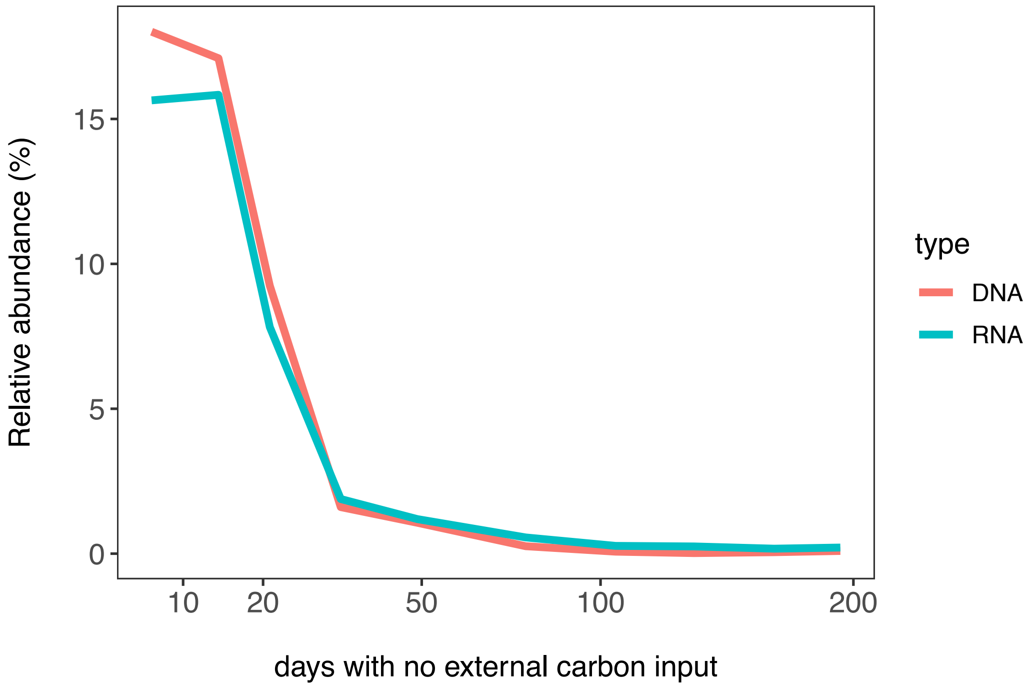

Figure S6. Temporal dynamics of Alteromonadales (now Enterobacterales) relative abundance in the 16S DNA-based community and the 16S RNA (cDNA)-based community during a long-term starvation experiment (with no external organic carbon inputs). See Sebastián et al. (2018). EMI 20:713-723 for more details
